# Early Origin for Human-Like Precision Grasping: A Comparative Study of Pollical Distal Phalanges in Fossil Hominins

**DOI:** 10.1371/journal.pone.0011727

**Published:** 2010-07-22

**Authors:** Sergio Almécija, Salvador Moyà-Solà, David M. Alba

**Affiliations:** 1 Institut Català de Paleontologia, Universitat Autònoma de Barcelona, Cerdanyola del Vallès (Barcelona), Spain; 2 ICREA at Institut Català de Paleontologia and Unitat d'Antropologia Biològica (Dept. BABVE), Universitat Autònoma de Barcelona, Cerdanyola del Vallès (Barcelona), Spain; University at Albany, State University of New York (SUNY), United States of America

## Abstract

**Background:**

The morphology of human pollical distal phalanges (PDP) closely reflects the adaptation of human hands for refined precision grip with pad-to-pad contact. The presence of these precision grip-related traits in the PDP of fossil hominins has been related to human-like hand proportions (i.e. short hands with a long thumb) enabling the thumb and finger pads to contact. Although this has been traditionally linked to the appearance of stone tool-making, the alternative hypothesis of an earlier origin—related to the freeing of the hands thanks to the advent of terrestrial bipedalism—is also possible given the human-like intrinsic hand proportion found in australopiths.

**Methodology/Principal Findings:**

We perform morphofunctional and morphometric (bivariate and multivariate) analyses of most available hominin pollical distal phalanges, including *Orrorin*, *Australopithecus*, *Paranthropous* and fossil *Homo*, in order to investigate their morphological affinities. Our results indicate that the thumb morphology of the early biped *Orrorin* is more human-like than that of australopiths, in spite of its ancient chronology (ca. 6 Ma). Moreover, *Orrorin* already displays typical human-like features related to precision grasping.

**Conclusions:**

These results reinforce previous hypotheses relating the origin of refined manipulation of natural objects–not stone tool-making–with the relaxation of locomotor selection pressures on the forelimbs. This suggests that human hand length proportions are largely plesiomorphic, in the sense that they more closely resemble the relatively short-handed Miocene apes than the elongated hand pattern of extant hominoids. With the advent of terrestrial bipedalism, these hand proportions may have been co-opted by early hominins for enhanced manipulative capabilities that, in turn, would have been later co-opted for stone tool-making in the genus *Homo*, more encephalized than the previous australopiths. This hypothesis remains may be further tested by the finding of more complete hands of unequivocally biped early hominins.

## Introduction

**Figure 1:**
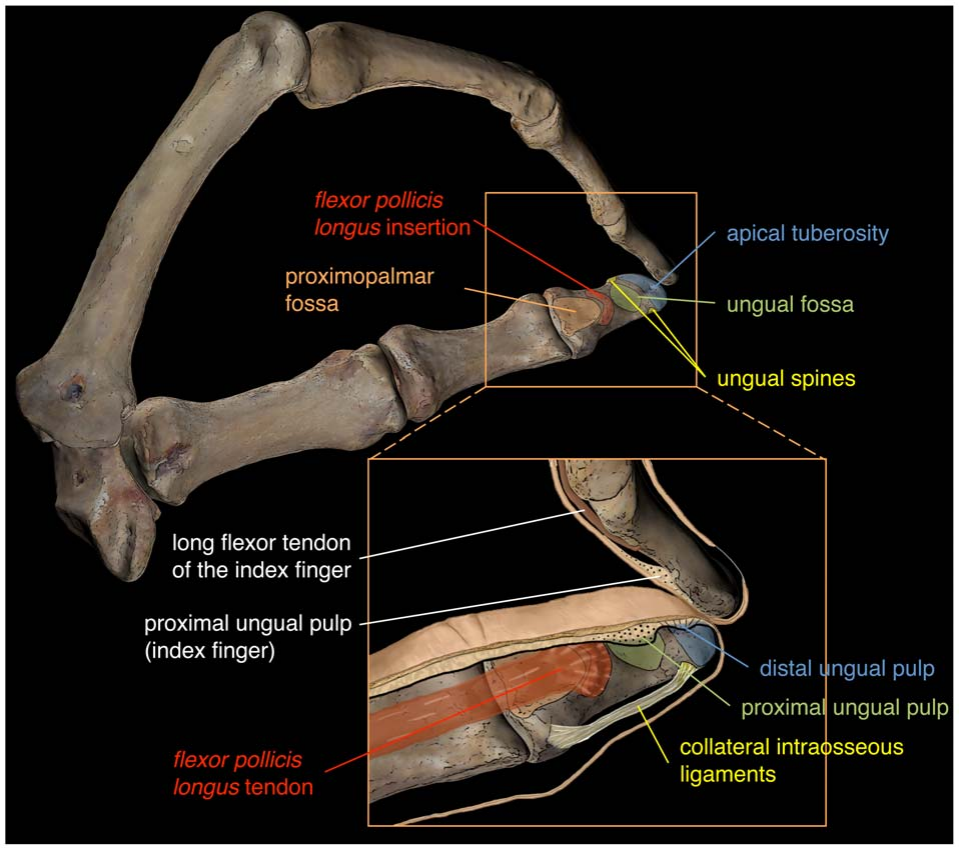
Modern human thumb and index finger (right hand) during pad-to-pad precision grasping in ulnar view. The box shows the anatomy of the pollical distal phalanx and its relationship with soft structures related to refined manipulation: a huge proximopalmar fossa (orange), associated with a palmarly protruding ridge (red) for insertion of the *flexor pollicis longus*; a compartmentalized digital pulp to accommodate the shape of the object being manipulated; this is reflected in the presence of an ungual fossa (green), associated to the large and mobile proximal pulp, as well as a wide apical tuberosity associated with the smaller and less mobile distal pulp; and finally, the ungual spines (yellow), where the collateral intraosseous ligaments that sustain the nail bed insert.

One of the hallmarks of humankind is the possession of a complex repertoire of manual grips [1]–[2]. In humans, the thumb always plays a central role, being involved in almost all possible prehensile typologies [1]–[4]. This is possible thanks to human intrinsic manual proportions, i.e. a long thumb relative to the rest of the hand. On the contrary, extant apes possess relatively long hands with a short thumb, in which the musculature is poorly developed [1]–[2], [5]. The most refined expression of human manipulation is attained during pad-to-pad precision grasping, which consists in the opposition of the proximal pulp of the thumb against that of one or more fingers ([3]; see Figure 1). This capability is reflected in the morphology of the distal phalanges, especially in the pollical distal phalanx (PDP), which shows specific features related to the soft tissues involved in precision grasping [3]–[4]. These include the pronounced insertion for the *flexor pollicis longus* (FPL), with a marked asymmetry towards the radial side; the presence of an ungual fossa; and the occurrence of dissymmetric ungual spines, with a prominent ulnar one (Figure 1). The asymmetries of the FPL insertion and the ungual spines are the osteological correlates of the interphalangeal joint configuration of the human thumb, in which flexion is accompanied by pronation, so that the pulp of the thumb faces that of the remaining fingers. This provides the maximum contact surface with the objects being manipulated. The presence in humans of ungual spines and ungual fossa are indicative of a fully compartmentalized digital pulp, with a fatty and mobile proximal portion (related to the ungual fossa) as well as a large and more or less static distal part (related to the distal tuberosity; [3]–[4]). The presence of these two different pulps, each with distinctive properties, ensures an adequate friction and accommodation of the object between the pulp of the thumb and those of the fingers during precision grasping (Figure 1). Furthermore, the possession of a wide apical tuberosity is correlated with the presence of pulp that is also wide [6].

**Figure 2:**
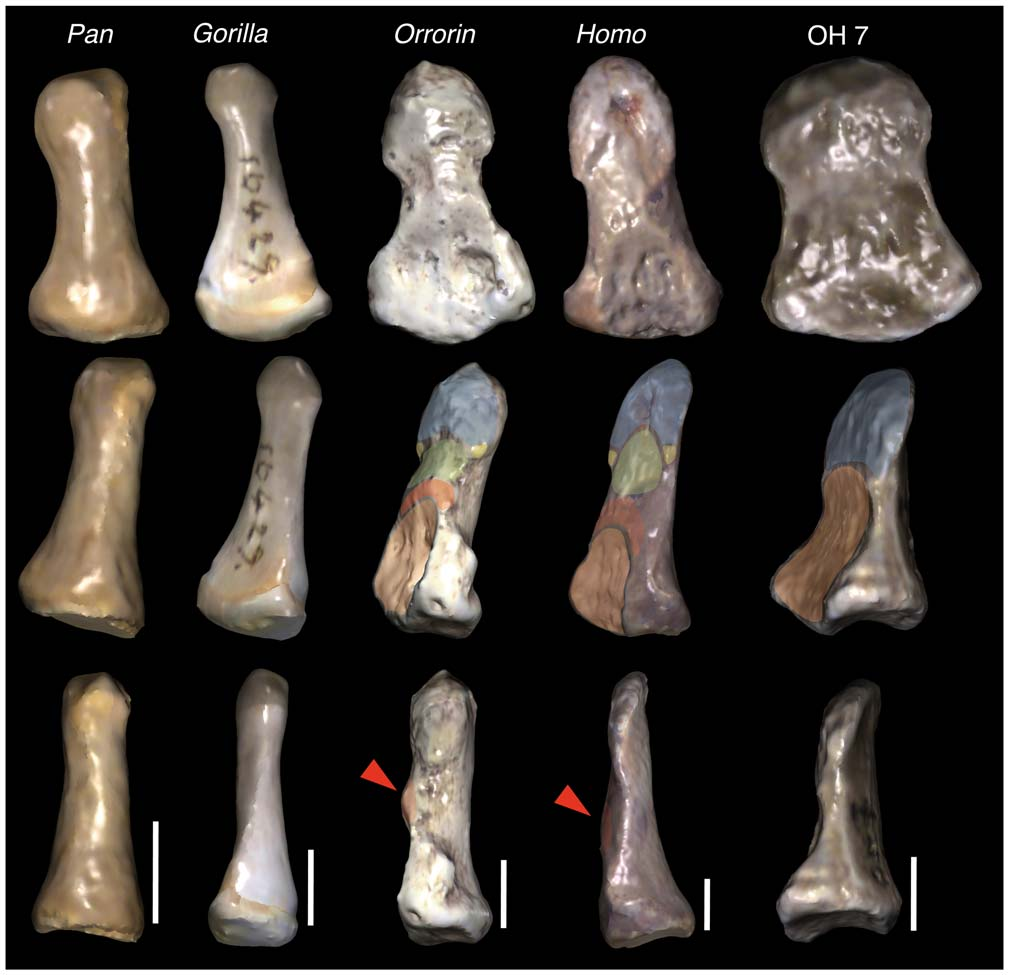
Morphological comparisons of pollical distal phalanges in African apes, extant humans and selected hominins. Specimens are showed in palmar (top), oblique proximopalmar (middle) and lateral (bottom) views, and scaled to the same length to easily visualize the morphological differences. The main features related to human-like precision grasping are indicated in the middle row (same colors as in Figure 1), whereas the palmarly protruding insertion for the *flexor pollicis longus* has been further signaled in lateral view (red arrows in the lower row). Note that, although with several morphological differences, all the features related to refined manipulation in modern humans are already present in the late Miocene *Orrorin*. By the way, the OH 7 specimen, besides its odd overall proportions, neither shows a distinctive insertion for the flexor muscle, nor a compartmentalized digital pulp. All the phalanges belong to a right thumb. Scale bars represent 5 mm.

Humans also display a characteristic FPL insertion, which protrudes palmarly as a distinct bony element that is visible in lateral view (Figures 1 and 2; [7] his Figure 4). Exclusively among extant primates, humans display the complete set of anatomical traits in their PDPs, which have been related to the presence of a relatively long and powerful thumb, able to contact the proximal pulp of the other fingers ([4]; Figure 1). Most previous studies have focused on the functional relationship between the anatomical traits discussed above and stone tool-making in Plio-Pleistocene hominins [3]–[4], [8]–[9]. Furthermore, some studies equated precision grasping—inferred from PDP anatomy— with stone tool-making, thus favoring the view that the evolution of the human hand was mainly related the selective pressures posed by the latter behavior [7], [10]–[11]. However, the human-like manual proportions displayed by australopiths [12]–[13], well before the advent of stone tool-making, would contradict the former hypothesis. In fact, ever since Darwin [14] it has been hypothesized that the origin of bipedalism was related to the freeing of the hands for manipulative purposes. Alternatively, manual proportions might have been optimized for manipulation once the hands became freed from locomotion thanks to the advent of terrestrial bipedalism [12], [15].

In order to test this hypothesis, and given the tight relationship between the anatomy of the PDP and refined manipulation in modern humans, we provide a morphofunctional analysis of this bone in selected fossil hominins—including the early biped *Orrorin tugenensis* (ca. 6 Ma; [16]–[17])—as compared to extant apes and humans. A principal components analysis (PCA) based on shape variables of the PDP is further provided in order to compare the main proportions of this bone in extant great apes, humans and fossil hominins, as well as ratios of relative phalangeal robusticity. The presence of anatomical traits functionally related to pad-to-pad precision grasping in the PDPs of early hominins would suggest that these taxa also displayed human-like hand length proportions for enabling the contact between the pulps (or pads) of the thumb with those of one ore more of the remaining fingers [4]. However, more complete fossil hands of these early hominins would be required in order to unequivocally confirm this prediction. On the contrary, the latter would be falsified if fossil taxa displaying the refined manipulation traits on their PDPs together with relatively long hands and short thumbs were found in the future.

## Results

### Precision grasping morphology

Extant great-ape PDPs lack all the features related to precision grasping (Figure 2); as such, they display a smooth apical tuft (instead of a developed apical tuberosity with ungual spines), and further lack well-developed basal tubercles, which in humans reflect the presence of collateral intraosseous ligaments for sustaining the nail bed ([3]–[4]; see Figures 1 and 2 and Videos S1, S2, S3, S4, S5 for renderings of the PDPs in Figure 2).

Interestingly, the PDP of *Orrorin*, being the earliest pollical specimen in the hominin fossil record (ca. 6 Ma; [16]–[17]) displays an overall human-like morphology (Video S3). The latter even includes the most significant features related to precision grasping (Figure 2), although some of them (such as the ridge for insertion of the FPL and the apical tuberosity) are stouter as compared to later hominins and modern humans [17].

The Olduvai Hominid 7 (OH 7) specimen—originally attributed to *Homo habilis* [17]—differs from that of extant apes and humans (Video S5). This PDP does not display ungual spines [3], and there is no ridge for insertion of the FPL, but a huge palmar fossa that extends until the large apical tuberosity (Figure 2). Furthermore, the lack of a distinctive ungual fossa and spines would be indicative of limited palmar pad compartmentalization and, as such, of a restricted precision-grip capability [3].

### PCA

**Figure 3 pone-0011727-g003:**
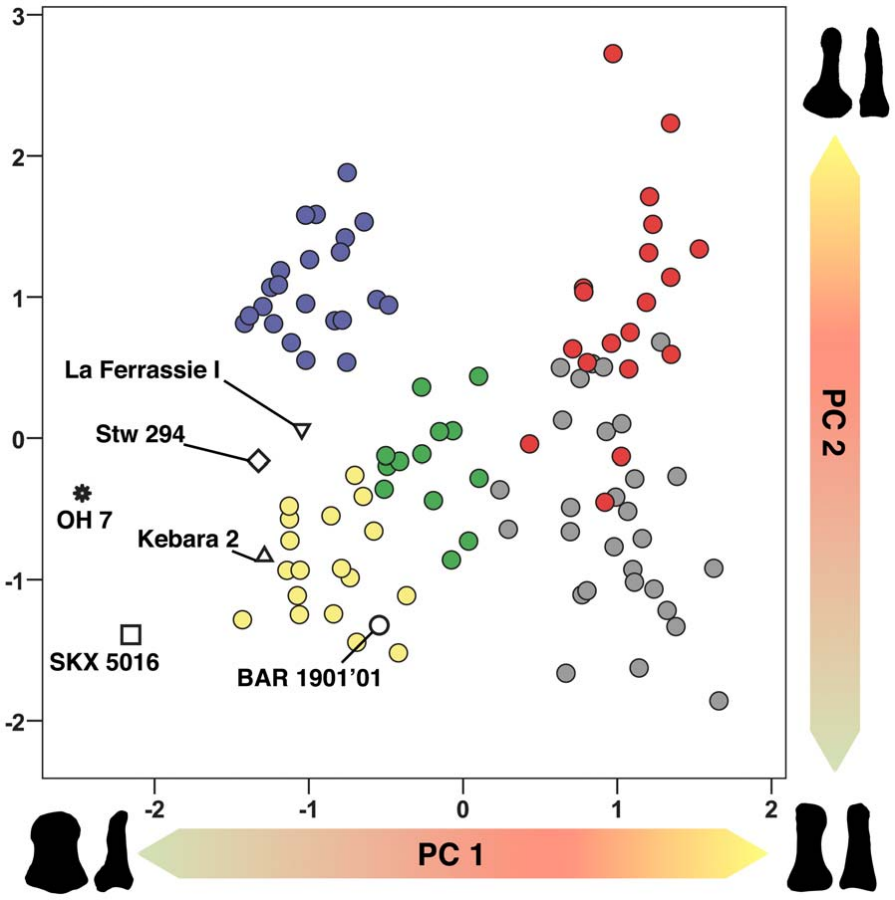
Principal components analysis (PCA) based on six shape variables of the pollical distal phalanx. Blue, *Papio*; red, *Pongo*; yellow, *H. sapiens*; green, *Gorilla*; grey, *Pan*. The PC 1 largely reflects the proportions of the tuft and shaft, while the PC 2 is more related to the proportions of the base. The *Orrorin* PDP overlaps with modern humans in both principal components, and later hominins also resemble modern humans in both components—although to a lesser degree. *Paranthropus robustus* and OH 7 constitute an exception, because they fall within the human range across the PC 2, but depart from the remaining taxa on the PC 1 by showing exceptionally wide PDPs (Figures 2 and 3). See text for further explanation. Figures at the corners represent the outline of these phalanges in palmar and lateral views.

A PCA based on PDP shape variables allow us to discriminate the several extant genera being analyzed between each other (Figure 3, see Materials and Methods and Table 1). Positive values on the PC 1 (68% variance) are related to phalanges with mediolaterally narrow tufts and shafts, and with dorsopalmarly high midshafts and bases, thus having an overall rod-like appearance. Negative values, on the contrary, are related to phalanges with a flat appearance due to high breadths at midshaft and at the distal end (i.e. with apical tuberosities instead of tufts). Positive values on the PC 2 (13% variance) mainly separate phalanges with a relatively large base, in both mediolateral and dorsopalmar diameters (i.e., with a relatively small shaft and apical tuft), from phalanges that are very long relative to other dimensions (see Figures 2 and 3 and Videos S1, S2, S3, S4, S5).

**Table 1:** Main results of the principal components analysis (PCA) based on the six shape variables of the pollical distal phalanx, including the variable loadings from the rescaled component matrix for the five principal components.

	PC 1	PC 2	PC 3	PC 4	PC 5
**% variance**	68.668	13.344	8.072	7.149	2.768
**% cumulative variance**	68.668	82.012	90.084	97.232	100
**Eigenvalue**	0.67	0.13	0.08	0.07	0.03

	Variable loadings				
Variables	PC 1	PC 2	PC 3	PC 4	PC 5
**L**	**0.837**	**−0.483**	0.111	0.230	−0.027
**MLT**	**−0.912**	−0.006	−0.320	0.256	0.000
**DPS**	**0.847**	0.042	−0.393	−0.347	0.077
**MLS**	**−0.886**	−0.275	0.201	−0.307	−0.064
**DPB**	0.520	**0.744**	0.181	0.074	−0.372
**MLB**	−0.114	**0.602**	0.553	0.149	0.544

The more significant values across PC 1 and 2 (in bold) are discussed in the text.

Abbreviations: PC  =  principal component; L  =  length; MLT  =  mediolateral width at the tuft; DPS  =  dorsopalmar height at midshaft; MLS  =  mediolateral width at midshaft; DPB  =  dorsopalmar height at the base; MLB  =  mediolateral width at the base. The sixth component was not included because the first five components almost explained the 100% of the total variance.

To this respect, extant great apes display relatively narrow PDPs with a dorsopalmarly high midshaft, while the apical tuft is not well developed, conferring them a rod-like shape. This is especially true concerning *Pan* and *Pongo*, which can be roughly distinguished from each other thanks to the highest loads on PC 2 of orangs, which display very small shafts and apical tufts relative to the base (Figure 3). On this basis, gorillas occupy a central position on the scatter plot, because their PDPs overall resemble a somewhat flatter version of chimps' (see also Figure 2).

Extant humans display PDPs with relatively wide shafts and apical tuberosities (see Figure 2), as indicated by the low loads on the PC1. Moreover, they show low values on the PC2, which are also correlated to a significantly long PDP (Table 2). This combination of relatively long and wide PDP is exclusive of humans among the extant taxa analyzed (Figure 3). It is interesting to point out that, although humans and gorillas overlap in both PC 1 and PC 2, they occupy different regions in the morphospace.

**Table 2:** Descriptive statistics for the ratios of distal phalangeal robusticity.

DP1 MLT/L	Taxon	N	Mean	SD	95% CI		Range	
	***Pan***	33	0.268	0.039	0.254	0.281	0.197	0.336
	***Gorilla***	15	0.335	0.032	0.317	0.353	0.276	0.392
	***Pongo***	23	0.273	0.030	0.260	0.286	0.227	0.359
	***Homo***	35	0.407	0.045	0.391	0.422	0.316	0.515
	***Papio***	22	0.478	0.050	0.456	0.501	0.391	0.571
	***Macaca***	18	0.494	0.084	0.453	0.536	0.354	0.620
	**OH 7**	1	0.611					
	***P.robustus***	1	0.566					
	**La Ferrassie I**	1	0.485					
	**Kebara 2**	1	0.456					

DP3 MLT/L	Taxon	N	Mean	SD	95% CI		Range	
	***Pan***	32	0.313	0.056	0.292	0.333	0.187	0.421
	***Gorilla***	15	0.385	0.047	0.359	0.410	0.316	0.480
	***Pongo***	26	0.229	0.024	0.219	0.239	0.184	0.268
	***Homo***	21	0.403	0.050	0.380	0.426	0.300	0.470
	***Papio***	22	0.323	0.033	0.309	0.338	0.253	0.371
	***Macaca***	14	0.294	0.069	0.254	0.334	0.218	0.423
	**OH 7**	1	0.437					
	***P.robustus***	1	0.397					
	**La Ferrassie I**	1	0.467					
	**Kebara 2**	1	0.426					

DP1MLT/L - DP3MLT/L	Taxon	N	Mean	SD	95% CI		Range	
	***Pan***	32	−0.043	0.043	−0.059	−0.028	−0.134	0.049
	***Gorilla***	12	−0.052	0.049	−0.084	−0.021	−0.145	0.028
	***Pongo***	21	0.045	0.036	0.028	0.061	0.005	0.175
	***Homo***	20	0.004	0.037	−0.014	0.021	−0.072	0.057
	***Papio***	22	**0.155**	0.052	**0.132**	**0.178**	0.067	0.301
	***Macaca***	14	**0.205**	0.081	**0.158**	**0.252**	0.077	0.347
	**OH 7**	1	**0.174**					
	***P.robustus***	1	**0.169**					
	**La Ferrassie I**	1	0.017					
	**Kebara 2**	1	0.030					

The more important values of relative robusticity (in bold) are discussed in the text.

Abbreviations: DP1  =  pollical distal phalanx; DP3  =  middle finger distal phalanx; L  =  length; MLT  =  mediolateral width at the tuft; SD  =  standard deviation; CI  =  confidence interval for the mean.

The position of baboons (*Papio*) in the scatter plot indicates that they show overall proportions on PDPs more similar to those of humans than to great apes, although being relatively shorter and displaying a larger base (Figure 3).

Concerning the fossil hominins, *Orrorin* (BAR 1901′01) most closely resembles modern humans in both components (Figure 3). Neandertals (La Ferrassie I and Kebara 2) and Stw 294 (cf. *A. africanus*) also fall close to modern humans in both components, although both La Ferrassie I and Stw 294 display slightly highest values on the PC 2. However, both *Paranthropus robustus* (SKX 5016) and OH 7 depart in PC 1 by having an extremely robust (i.e mediolaterally broad) shaft and apical tuberosity in the PDP.

### Ratios

**Figure 4:**
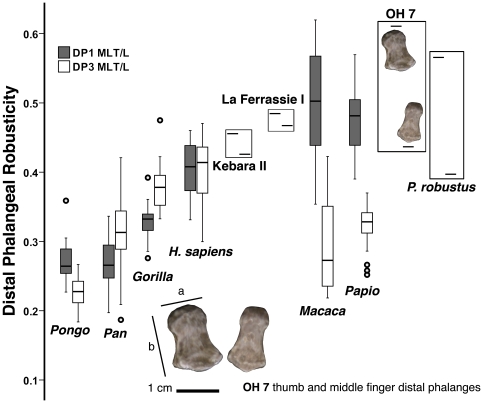
Boxplots of distal phalangeal robusticity in selected extant taxa, Neandertals, OH 7 and *Paranthropus robustus*. Robusticity refers to apical tuft width (a) in relation to maximum length (b) of the distal pollical and middle finger phalanges (left and right, respectively; see Materials and  for further details). Horizontal lines represent the median values, whereas the boxes represent the 25% and 75% percentiles, the whiskers the maximum-minimum ranges and circles are outliers. OH 7, like *Paranthropus robustus*, display a robusticity pattern convergent with quadrupedal monkeys (*Macaca* and *Papio*), in which the pollical distal phalanx is disproportionally robust relative to that from the middle finger. Note that the pollical and nonpollical distal phalanges attributed to *P. robustus* may not belong to the same individual.

Here we provide additional morphometric evidence regarding the robusticity of the distal phalanges, by comparing the first (DP1) and third (DP3) manual rays of extant taxa, together with OH 7, *Paranthropus robustus* and Neandertals (Figure 4 and Table 2). In orangutans, the PDP is only slightly more robust than the third distal one, whereas in African apes the reverse condition is found. The 95% confidence intervals (CI) for DP1 and DP3 robusticity between extant great apes do not overlap, suggesting that differences are significant. In modern humans the degree of distal phalanx tuft robusticity for the first and third manual rays is very similar, although it is somewhat higher in the thumbs of Neandertals, with both La Ferrassie I and Kebara II having robusticity values above the 95% CI of extant humans. In monkeys, on the contrary, the distal pollical phalanx is much more robust than the distal third one, with both macaques and baboons showing values well above the 95% CI to that of the third digit. This odd condition is also found in both OH 7 and *P. robustus*, which the difference between the robusticity of the two digits (DP1 - PD3) falling within the 95% CI displayed by monkeys (see Figure 4 and Table 2).

## Discussion

### Functional analysis

Extant great apes do not significantly use the thumb during locomotion: it does not participate in below-branch suspensory behaviors, and it does not support body weight during knuckle-walking in African apes [18]–[21]. However, it can participate during terrestrial palmigrady and fist-walking in orangutans [18]. In the latter, the hallux is reduced [22], and in some cases the distal phalanx may not be present at all [23]. This is explained by the specialized, four-digit hook grasp of orangs' hands and feet. According to this, the hallux would have been extremely reduced because of locomotor selection pressures, whereas the reduction of the pollex would not have proceeded to the same extent due to contradictory, manipulatory selection pressures favoring instead the possession of a longer thumb [23]. In orangutans, the reduction of the hallux has also affected the long flexor tendon [22]. This condition that can be also found in the thumb of all extant great apes, especially in *Pongo* and *Pan*, in which locomotor selection pressures have probably favored the lengthening and increased strength of digits II-V [18].

Thus, although extant great apes display diminished thumbs, especially concerning extrinsic muscle insertions, they do have well-developed intrinsic muscles that enable efficient power and precision grips, the latter being used during food manipulation [1], [2], [5], [18], [24]. Chimps and gorillas do efficiently manipulate small object between their thumb and index finger using different precision grip combinations (e.g. tip-to-tip and pad-to-side), but a human-like, pad-to-pad precision grip is precluded due to the disproportionate length of their digits II-V relative to that of the thumb [1], [2], [5], [24]. Chimps and orangs mostly rely on arboreal foraging by directly putting the foods from branch to mouth, whereas gorillas spend many hours on the ground, where they carefully select, manipulate and hold the food with their hands [18]. Increased terrestriality in such a large ape might have resulted in longer thumbs relative to the rest of the hand [12], [25], as a by-product of their shorter hands relative to body mass [26]. The *Gorilla* hand therefore displays more balanced proportions between the thumb and index finger (presumably related to somewhat advanced manipulatory capabilities) than chimps and especially orangs [5].

Our results show that extant great apes exhibit rod-like PDPs with barely discernible muscular impressions on the palmar side, further lacking developed tufts and associated palmar pads (Figures 2–4; see also [4], [6], [22]). The lack of dorspalmar flattening in the PDP, especially in *Pan* and *Pongo*, would be related to the lack of a developed flexor apparatus (usually without receiving extrinsic muscular component). The overall reduced PDP of orangs, especially referring the shaft and apical tuft, obviously stems from their rudimentary thumbs, which are diminished like in *Colobus* and *Procolobus* [22]. In the latter taxa, the PDP, when present, is usually confined to its proximal portion, the base (S.A. personal observation). Gorillas, being the most terrestrial great apes and displaying relatively short and skillful hands with relatively long thumbs, possess a somewhat flatter PDP than the other great apes (Figures 2–3). Furthermore, gorillas also exhibit more barely evidence of muscular insertion scars on the palmar side of the PDP shaft than them (Figure 2). Since the thumbs of gorillas do not participate in locomotion, this morphology should be correlated with an increased use of the thumb for manipulation as compared to *Pan* and *Pongo* [18].

Terrestrial monkeys, in its turn, exhibit proportionally short fingers in relation to their thumbs, thereby enabling an efficient precision grip [27]–[28]. This is particularly evident in gelada baboons, which display enhanced manual feeding capabilities [29] thanks to displaying the highest opposability index among extant primates, including humans [28]. Other baboons (*Papio* spp.) show the same capabilities, although to a lesser degree [28]. These baboons are digitigrade, and only the tip of the thumb contacts the substrate during the touchdown phase [21]. A huge long flexor tendon is inserted onto the distal part of the shaft and tuberosity of their PDP [4], although it does not correspond to the FPL, but to the radial portion of the *flexor digitorium profundus* [21]. Moreover, during the tip-weight support, their PDPs can be hyperextended, so that, like in humans, their pollical interphalangeal joint displays well-developed sesamoid bones [4]. There are also other similarities between baboon and human PDPs [4], such as a broad distal pad, tuberosity (sometimes with spines) and nail, a large palmar fossa (but not a distinct ridge for insertion of the FPL and ungual fossa), and similar ratios concerning both bone and long flexor tendon dimensions. These similarities can be related to the enhanced manipulative capabilities displayed by these monkeys [27]–[29].

Our results agree with previous findings showing that baboons display PDPs more similar in overall proportions to those of extant humans rather than those of extant great apes, although being shorter and displaying a larger base (Figure 3). This morphology might be an adaptation to frequent weight bearing, during which the tip of the thumb contacts the ground in hyperextended postures. According to this, the enhanced manipulative capabilities displayed by baboons could be merely a by-product of an adaptation of the hand to digitigrady, resulting in a long thumb relative to the rest of the hand that would be suitable for pad-to-pad precision grasping (see Fig. 8a–b in [28]). The precision grasping displayed by these monkeys, in any case, is much less developed than that of humans, which is further reflected by the lack in the former of many PDP traits that are functionally related to human-like precision grasping (Figures 1–2; [3]–[4]).

The morphology displayed by the PDP of modern humans—including a relatively long bone, dorsopalmarly flat and wide at the shaft and apical tuberosity (Figure 3), together with several morphological traits related to a powerful FPL and compartmentalized digital pulp (Figure 2)—is not found among non-human primates, being indicative of a stable and powerful pad-to-pad contact during refined manipulation [3]–[4]. The similar position occupied by the PDPs of modern humans and those of fossil hominins (including Neandertals), together with the possession of morphological features functionally-related to pad-to-pad precision grasping [17], [30]–[31], is highly indicative of shared functional similarities. Apart from Neandertals, this is evident in Stw 294 (cf. *A. africanus*) and, particularly, in BAR 1901′1 (*Orrorin*), the latter showing, among early hominins, the greatest resemblance to extant humans (Figures 2–3). On the contrary, SKX 5016 (*P. robustus*) and OH 7, although more closely resembling extant humans than any other taxa examined, display extremely broad shafts and apical tuberosities (Figures 3–4). Although the phalanx attributed to *P. robustus* has been described as displaying some traits related to pad-to-pad precision grasping [17], this is not the case of OH 7 [3]–[4], which lacks some of these features (Figure 2 and Video S5; see next section). These results agree with previous ones, according to which both OH 7 and SKX 5016 are extremely broad phalanges, unlike those of humans or apes, thus having a narrow base relative to these dimensions [32].

All the evidence reported above suggests that the morphological differences found between the PDPs of extant taxa seem to be also correlated to functional differences in hand function, particularly involving the thumb. It would be of utmost interest to discern whether the subtle morphological differences found amongst the fossil hominins being analyzed—particularly concerning SKX 5016 and OH 7—are also correlated to differences in function, or whether they are alternatively correlated to different overall body types [32]. This cannot be definitively settled until more postcranial remains of these hominins are available. However, given the apparently convergent morphological similarities between these fossil taxa and baboons, both in the middle [33] and distal phalanges [this study], we favor the view that some kind of functional differences as compared to other hominins are likely.

### The attribution of the OH 7 hand remains

The original attribution of the partial hand from Olduvai Bed I to the holotype of *H. habilis* (OH 7, consisting in the parietals and mandible of a subadult; [34]) has been subsequently accepted by most authors [e.g. 2]–[3], [35]–[36], mainly due to its subadult status. However, due to its overall robusticity—especially concerning the PDP—and curved middle phalanges, an alternative attribution to *Paranthropus*—contemporary to *Homo* in that region [37]—was also suggested long ago [18], [38]. More recently, this alternative attribution has been further favored on the basis of morphometric and morfofunctional analyses, particularly concerning middle phalangeal morphology [33]. The later study found that the OH 7 remains are more similar to those from South Africa attributed to *P. robustus* than to earlier *Australopithecus* and later and contemporary humans [33]. It is also noteworthy that other bones from the OH 7 hand, such as the trapezium, also suggests a taxonomic attribution different than *Homo* [9].

Be that as it may, the odd morphology of OH 7 PDP led some authors to consider an alternative anatomical identification as a hallucial distal phalanx [35]. This would be supported by the strong muscular attachments and, especially, the slight axial torsion at the apical tuft (functionally related to bipedalism). However, a discriminant analysis by the same authors indicated “that the fossil is closer to human distal phalanges than to those of any other hominoid and is somewhat more like a pollical than a hallucial phalanx” [35, p. 325]. We concur with this anatomical identification, especially given that there are no differences in axial torsion as compared to modern humans or extant apes (Figure 2), in which the apical tuft is slightly twisted, so that it faces the rest of the fingers. It is also noteworthy that the PDP of OH 7 does not display a human-like morphology related to precision grasping ([3]; Figure 2 and Video S5). This includes its odd overall proportions, which like in SKX 5016 (*P. robustus*) are characterized by a high degree of apical and midshaft robusticity, as it is found here (Figure 3) and in many previous works [10], [32]–[33], [39].

Furthermore, our results regarding relative distal phalangeal robusticity show that, when non-pollical manual rays are also taken into account, the pattern of robusticity of OH 7, like that of *Paranthropus*, resembles that of quadrupedal monkeys and does not fit either the great-ape or the human pattern (Figure 4). These results agree with a previous study of this partial hand, which interpreted the morphology of the middle phalanges as showing convergent adaptations with gelada baboons [33]. Alternatively, it would be necessary to conclude that early *Homo* was more similar to *Paranthropus* than to *Australopithecus* and *Orrorin* regarding several aspects of phalangeal morphology, which in evolutionary terms would imply a reversion regarding, among others, the robusticity of the PDP.

### The attribution of BAR 1901′01

Hallucial distal phalanges do not display the set of features present in all the human PDPs, which are related to the pad-to-pad contact. Thus, they do show a large proximoplantar fossa, which is further accompanied by plantarly protruding basal tubercles. In proximal view, these structures configure a very wide and shallow channel for the pass and/or insertion of the *flexor hallucis longus*, which can act as a “supporting muscle” during terrestrial progression in both apes and humans [40].

Thus, besides morphometric similarities, the PDP of the late Miocene *Orrorin* displays the typically human set of morphological features functionally related to human pad-to-pad precision grasping (Figure 2 and Videos S3–S4; [4]). The presence of these features, among others, indicates that BAR 1901′1 does not belong to the hallux. The other features indicating an anatomical attribution to the pollex include: its degree of elongation, overall flatness, and round dorsal surface [17]; the orientation of the apical tuberosity, which does not face distally (such is the case in distal phalanges that support weight stresses in hyperextended positions; see Fig. 1 in [41] for the case of OH 10); and the lack of axial torsion, which is present in the hallucial phalanges of both humans [41] and apes [42].

### The evolution of refined manipulation

The presence of precision grip features in the PDP of *Orrorin* indicates that this bone was fully prepared to accommodate objects between the palmar aspect of its pulp and that of the fingers. Some of these precision-grasping features in the *Orrorin* specimen had been previously reported, although they were interpreted as an adaptation to arboreal locomotion reflecting “the precision grip essential for climbing and balancing, different from that of apes” ([17], p. 372). However, given the fact that no arboreal primate displays this set of features, we favor the hypothesis that functionally relates the striking and detailed similarities between *Orrorin* and extant human PDPs to refined object manipulation.

Admittedly, although the *Orrorin* phalanx mostly looks like a human PDP, it also displays some primitive features that are further retained by the later australopiths, such as a small ungual fossa and a proximally protruding median eminence of the articular surface. Moreover, some other features, like the ridge for insertion of the FPL, and the dorsopalmar height of the shaft and apical tuberosity, are stouter than in later hominins and modern humans (Figure 2). The latter display relatively flat PDPs, especially at midshaft and distal tuberosity levels (Figures 2–3). Thus, although the late Miocene *Orrorin* is somewhat ape-like in dorsopalmar dimensions, it approaches a human-like profile in mediolateral dimensions. Moreover, it is most remarkable that although Plio-Pleistocene australopiths also show the morphological features related to precision grip [17], the 6-million-years-old PDP of *Orrorin* is more human-like in overall proportions and morphology than these later hominins. To this respect, both OH 7 and SKX 5016 display a degree of mediolateral broadening that highly surpasses the human condition. This is consistent with the femoral morphology of this taxon, which more closely resembles australopiths than later *Homo*, but among early hominins it is the one that most closely resembles humans [43]. All this evidence suggests that australopiths—especially *Paranthropus*—are derived by displaying a robusticity pattern on the distal phalanges that is convergent with that of quadrupedal monkeys (Figure 4), as previously suggested for the middle phalanges [33], and also suggested to some degree by the trapezial morphology [9], [36].

Be that as it may, the highest resemblance between the late Miocene *Orrorin* and modern humans, with the exclusion of australopiths, is an unexpected result that bears important implications for the understanding of the selective pressures originally involved in the evolution of human manual skills. Extant great apes are highly committed to arboreal locomotion (including vertical climbing and suspensory behaviors), their manipulative capabilities being limited by their relative short thumbs [1]–[2], [5], and especially by their absolutely long hands [12]. Because Miocene apes displayed absolutely long thumbs, it has been suggested that their hands were more suitable for manipulative purposes than those of extant hominoids [44]–[47]. The same condition can also be inferred for the stem hominid *Pierolapithecus*, which displays relatively short manual phalanges—like other early and middle Miocene apes—as well as a relatively long PDP [48]–[49].

From the evidence presented above, it can be suggested that the hand length proportions of humans are plesiomorphic to a large extent. If *Orrorin* was an early biped hominin with a PDP adapted to refined manipulation, it follows that the whole thumb would be long relative to the hand, thus enabling an efficient pad-to-pad contact. Thus, this leads us to hypothesize that the manual proportions of *Orrorin* might have more closely resembled those of early and middle Miocene apes than those of extant apes, which would have diverged towards a different direction from the same ancestral morphotype. This idea is similar to that presented by Tuttle [18], who suggested that “By the time early hominids had assumed a bipedal gait, the hand was probably well on its way toward modern human configuration” [18; p. 203]. We therefore favor the hypothesis that human hand proportion enabling a pad-to-pad precision grasping are an exaptation, co-opted by early bipedal hominins for manipulative purposes, but originally evolved within a locomotor selective context as an adaptation to powerful-grasping—assisted by the thumb— within an arboreal setting in Miocene apes. This would explain why early bipedal hominins, such as *A. afarensis* [12] and *A. africanus* [13], already displayed human-like hand length proportions prior to the appearance of stone tools in the archeological record. It seem likely that the acquisition of habitual bipedalism—which largely freed the upper extremities from locomotor demands [14]—would have facilitated the refining of the manipulative capabilities displayed by all primates [e.g. 1]–[2], [5], [12], [28], [33]. Furthermore, bipedalism could also affect the hand morphology by means of correlated developmental responses dues to changes in the feet morphology [12], [39]. Thus, most probably “early hominoids [and hominins] […] evidently employed behaviors resulting from the addition of a number of functional morphological innovations to a relatively conservative body plan, including those underlying a more sophisticated manipulative and grasping use of the hand than used before” [50, p. 264].

Most likely, these hand capabilities would have not been co-opted until much later for a regular use in stone tool-making, coinciding with the encephalization increase that took place in hominins with the advent of the genus *Homo* ca. 2.5 Ma [51]–[53]. Thus, stone tool-making would not have played a significant role until the latter part of human hand evolution, and especially after the advent of the Acheulian culture [54], as already suggested by Tuttle [18]. On the basis of the evidence provided by modern human hands, the fine tuning of manipulative adaptations during the evolution of *Homo* would have involved an increase of overall robusticity at the several manual joints, an increase in robusticity of the whole thumb, and especially the development of distinct palmar pads on the distal phalanges.

More complete fossil hands from the African Mio-Pliocene transition would be necessary to clarify this issue, especially regarding of the selection pressures underpinning the remarkable human-like features of the *Orrorin* PDP. For the moment being, the most complete fossil hand around this time corresponds to the 4.4 million-years-old putative hominin *Ardipithecus ramidus* [55]. Although its hand is not as elongated as in extant apes [55], its thumb seems to be relatively short, and its PDP looks more ape-like than that of *Orrorin*, in spite of the older chronology of the latter. Thus, although *A. ramidus* has been claimed to be an early hominin close to the last common ancestor with *Pan* [56], it could be alternatively interpreted as one of the apes “among the tangled branches that constitute the basal hominine bush” [57, p 533]. Only future comparative studies would help to bring some light onto this question.

### Conclusions

The pollical distal phalanx of the early bipedal hominin *Orrorin* (BAR 1901′01) unequivocally shows precision grasping capabilities in spite of its ancient chronology, most closely resembling modern humans than some later Plio-Pleistocene hominins—which show a derived robusticity pattern. This indicates that refined manipulation is an ancient acquisition already present by the late Miocene. This is consistent with the hypothesis that habitual terrestrial bipedalism and the possession of skillful hands do constitute a single adaptive complex. Both types of behaviors might have been simultaneously selected, by synergistically favoring each other.

From the evidence reported by BAR 1901′01, it is reasonable to assume that human hand length proportions (i.e. short hands and relatively long thumbs) are plesiomorphic to some degree, thus more closely resembling the short hands with relatively long thumbs of Miocene apes, rather than the elongated hands of extant apes, which seem to be secondarily derived. These ancient proportions, suitable for refined manipulation, would not have been co-opted for stone tool-making until much later, coinciding with a significative increase in encephalization in the genus *Homo*.

## Materials and Methods

### The primate sample

The comparative extant sample includes the following taxa: chimpanzees and bonobos (*Pan*; N = 29), gorillas (*Gorilla*; N = 13), orangutans (*Pongo*; N = 19), baboons (*Papio*; N = 22) and modern humans (*Homo sapiens*; N = 22). The fossil sample includes the PDPs of *Orrorin tugenensis* from the Lukeino Formation (BAR 1901′01), *Australopithecus africanus* from Sterkfontein (Stw 294), the hand from Olduvai (OH 7 A), *Paranthropus robustus* from Swartkrans (SKX 5016), and *H. neanderthalensis* from La Ferrassie I and Kebara 2. Apart of the above-mentioned extant taxa, we further employed the following fossil third distal phalanges for computing ratios: OH 7 B, SKX 27504 (attributed to *P. robustus*), La Ferrassie I and Kebara 2. Measurements for fossil specimens were taken from originals, good quality casts or from the literature [31], [58]–[59].

### PCA

A principal components analysis (PCA) based on the covariance matrix was employed to perform morphometric comparisons between the pollical distal phalanges (PDP) of selected fossil specimens and those of other hominins and extant primates, including modern humans. This analysis, which does not assume group membership on a priori grounds, was based on six shape variables of the PDP, in its turn computed on the basis of the following six metrial variables: length (L); mediolateral width at the apical tuft (MLT), midshaft (MLS) and the base (MLB); and dorsopalmar height at midshaft (DPS) and the base (DPB). These measurements were transformed into shape variables by dividing each of them by the geometric mean (GM) of all the six phalangeal measurements (the GM being taken as a variable of overall phalangeal size) and then applying a logarithmic transformation (on the basis of natural logarithms, ln), following [43].

### Ratios

Distal phalangeal robusticity was analyzed by means of a ratio between apical tuft width and maximum length, separately for both the distal pollical and middle finger phalanges. We further calculated the difference between both ratios, in order to quantify the relative robusticity of the pollical distal phalanx in relation to that of the middle finger. Summary statistics for extant and fossil taxa are reported in Table 2.

## Supporting Information

Video S1 360° video render of *Pan troglodytes* pollical distal phalanx.
(3.31 MB AVI)

Video S2 360° video render of *Gorilla gorilla* pollical distal phalanx.
(2.96 MB AVI)

Video S3 360° video render of *Orrorin tugenensis* pollical distal phalanx.
(3.72 MB AVI)

Video S4 360° video render of *Homo sapiens* pollical distal phalanx.
(3.46 MB AVI)

Video S5 360° video render of Olduvai Hominid 7 pollical distal phalanx.
(4.32 MB AVI)

## References

[1] Napier JR (1960). Studies of the hands of living primates.. The Proceedings of the Zoological Society of London.

[2] Napier J (1993). Hands [Revised by Russell H. Tuttle]..

[3] Shrewsbury MM, Sonek A (1986). Precision holding in humans, non-human primates, and Plio-Pleistocene hominids.. Human Evolution.

[4] Shrewsbury MM, Marzke MW, Linscheid RL, Reece SP (2003). Comparative morphology of the pollical distal phalanx.. American Journal of Physical Anthropology.

[5] Tuttle RH (1969). Quantitative and functional studies on the hands of the Anthropoidea.. Journal of Morphology.

[6] Mittra ES, Smith HF, Lemelin P, Jungers WL (2007). Comparative morphometrics of the primate apical tuft.. American Journal of Physical Anthropology.

[7] Susman RL (1998). Hand function and tool behavior in early hominids.. Journal of Human Evolution.

[8] Marzke MW (1997). Precision grips, hand morphology, and tools.. American Journal of Physical Anthroplogy.

[9] Tocheri MW, Marzke MW, Liu D, Bae M, Jones GP (2003). Functional capabilities of modern and fossil hominid hands: Three-dimensional analysis of trapezia.. American Journal of Physical Anthropology.

[10] Susman RL (1988). Hand of *Paranthopus robustus* from Member 1, Swartkrans: Fossil evidence for tool behavior.. Science.

[11] Susman RL (1994). Fossil evidence for early hominid tool use.. Science.

[12] Alba DM, Moyà-Solà S, Köhler M (2003). Morphological affinities of the *Australopithecus afarensis* hand on the basis of manual proportions and relative thumb length.. Journal of Human Evolution.

[13] Green DJ, Gordon A (2008). Metacarpal proportions in *Australopithecus africanus*.. Journal of Human Evolution.

[14] Darwin C (1871). The Descent of Man, and Selection in Relation to Sex..

[15] Hartwig WC, Doneski K (1998). Evolution of the hominid hand and tool making behavior.. American Journal of Physical Anthropology.

[16] Senut B, Pickford M, Gommery D, Mein P, Cheboi K (2001). First hominid from the Miocene (Lukeino Formation, Kenya).. Comptes Rendus de l'Académie des Sciences, Paris.

[17] Gommery D, Senut B (2006). La phalange distale du pouce d'*Orrorin tugenensis* (Miocène supérieur du Kenya).. Geobios.

[18] Tuttle RH (1967). Knuckle-walking and the evolution of hominoid hands.. American Journal of Physical Anthroplogy.

[19] Preuschoft H (1973). Functional anatomy of the upper extremity.. The Chimpanzee.

[20] Preuschoft H, Godinot M, Beard C, Nieschalk U, Jouffroy KK, Preuschoft H, Chivers DJ (1993). Biomechanical considerations to explain important morphological characters of primate hands.. Hands of Primates.

[21] Whitehead PF, Gebo DL (1993). Aspects of the anthropoid wrist and hand.. Postcranial Adaptation in Nonhuman Primates.

[22] Straus WL (1942). Rudimentary digits in primates.. The Quarterly Review of Biology.

[23] Tuttle RH, Rogers CM (1966). Genetic and selective factors in reduction of the hallux in *Pongo pygmaeus*.. American Journal of Physical Anthropology.

[24] Tuttle RH (1970). Postural, propulsive, and prehensile capabilities in the cheiridia of chimpanzees and other great apes.. The Chimpanzee.

[25] Schultz AH (1930). The skeleton of the trunk and limbs of higher primates.. Human Biology.

[26] Moyà-Solà S, Köhler M, Alba DM, Casanovas-Vilar I, Galindo J (2004). *Pierolapithecus catalaunicus*, a new Middle Miocene great ape from Spain.. Science.

[27] Napier JR, Napier PH (1967). A Handbook of Living Primates..

[28] Etter HF (1973). Terrestrial adaptations in the hands of Cercopithecinae.. Folia Primatologica.

[29] Jolly CJ (1970). The seed-eaters: A new model of hominid differentiation based on a baboon analogy. Diverse Approaches in Human Evolution.

[30] Ricklan DE (1987). Functional anatomy of the hand of *Australopithecus africanus*.. Journal of Human Evolution.

[31] Ricklan DE (1988). A funcional and morphology study of the hand bones of early and recent South African hominids..

[32] Smith SL (1995). Pattern profile analysis of hominid and chimpanzee hand bones.. American Journal of Physical Anthropology.

[33] Moyà-Solà S, Köhler M, Alba DM, Almécija S (2008). Taxonomic attribution of the Olduvai Hominid 7 manual remains and the functional interpretation of hand morphology in robust australopithecines.. Folia Primatologica.

[34] Leakey LSB, Tobias PV, Napier JR (1964). A new species of the genus *Homo* from Olduvai Gorge.. Nature.

[35] Susman RL, Creel N (1979). Functional and morphological affinities of the subadult hand (O.H. 7) from Olduvai Gorge.. American Journal of Physical Anthropology.

[36] Marzke MW, Tocheri MW, Steinberg B, Femiani JD, Reece SP (2010). Comparative 3D quantitative analyses of trapeziometacarpal joint surface curvatures among living catarrhines and fossil hominins.. American Journal of Physical Anthropology.

[37] Leakey LSB (1959). A new fossil skull from Olduvai.. Nature.

[38] Robinson JT (1972). Early Hominid Posture and Locomotion..

[39] Smith SL (2000). Shape variation of the human pollical distal phalanx and metacarpal.. American Journal of Physical Anthropology.

[40] Keith A (1929). The history of the human foot and its bearing on orthopaedic practice: Being the Third H. O. Thomas Memorial Lecture given before the Medical Institution, Liverpool, May 11, 1928.. The Journal of Bone and Joint Surgery.

[41] Day MH, Napier JR (1966). A hominid toe bone from Bed I, Olduvai Gorge, Tanzania.. Nature.

[42] Nakatsukasa M, Kunimatsu Y, Nakano Y, Ishida H (2002). Morphology of the hallucial phalanges in extant anthropoids and fossil hominoids.. Zeitschrift für Morphologie und Anthropologie.

[43] Richmond BG, Jungers WL (2008). *Orrorin tugenensis* femoral morphology and the evolution of hominin bipedalism.. Science.

[44] Pilbeam DR, Rose MD, Badgley C, Lipschutz B (1980). Miocene hominoids from Pakistan.. Postilla.

[45] Begun DR, Teaford MF, Walker A (1994). Comparative and functional anatomy of *Proconsul* phalanges from the Kaswanga Primate Site, Rusinga Island, Kenya.. Journal of Human Evolution.

[46] Madar SI, Rose MD, Kelley J, MacLatchy L, Pilbeam D (2002). New *Sivapithecus* postcranial specimens from the Siwaliks of Pakistan.. Journal of Human Evolution.

[47] Nakatsukasa M, Kunimatsu Y, Nakano Y, Takano T, Ishida H (2003). Comparative and functional anatomy of phalanges in *Nacholapithecus kerioi*, a middle Miocene hominoid from northern Kenya.. Primates.

[48] Almécija S, Alba D, Moyà-Solà S (2009). *Pierolapithecus*, *Hispanopithecus* and the evolution of positional behavior in Miocene apes: perspectives from the hand [Abstract].. Journal of Vertebrate Paleontology.

[49] Almécija S, Alba DM, Moyà-Solà S (2009). *Pierolapithecus* and the functional morphology of Miocene ape hand phalanges: paleobiological and evolutionary implications.. Journal of Human Evolution.

[50] Rose MD (1992). Kinematics of the trapezium-1st metacarpal joint in extant anthropoids and Miocene hominoids.. Journal of Human Evolution.

[51] Alba DM (2010). Cognitive inferences in fossil apes (Primates: Hominoidea): does encephalization reflect intelligence?. Journal of Anthropological Sciences.

[52] McHenry H (1976). Early hominid body weight and encephalization.. American Journal of Physical Anthropology.

[53] Kappelman J (1996). The evolution of body mass and relative brain size in fossil hominids.. Journal of Human Evolution.

[54] Tocheri MW, Orr CM, Jacofsky MC, Marzke MW (2008). The evolutionary history of the hominin hand since the last common ancestor of *Pan* and *Homo*.. Journal of Anatomy.

[55] Lovejoy CO, Simpson SW, White TD, Asfaw B, Suwa G (2009). Careful climbing in the Miocene: The forelimbs of *Ardipithecus ramidus* and humans are primitive.. Science.

[56] White TD, Asfaw B, Beyene Y, Haile-Selassie Y, Lovejoy CO (2009). Ardipithecus ramidus and the paleobiology of early hominids.. Science.

[57] Harrison T (2010). Apes among the tangled branches of human origins.. Science.

[58] Susman RL (1989). New hominid fossils from the Swartkrans Formation (1979-1986) excavations. Postcranial specimens.. American Journal of Physical Anthropology.

[59] Vandermeersch B, Yosef OB, Vandermeersch B (1991). La ceinture scapulaire et les membres supérieurs.. Le squelette mousterien de Kébara 2.

